# A Simple Specific Functional Test for SCD: VEMPs to High Frequency (4,000Hz) Stimuli—Their Origin and Explanation

**DOI:** 10.3389/fneur.2020.612075

**Published:** 2020-11-20

**Authors:** Ian S. Curthoys, Leonardo Manzari

**Affiliations:** ^1^Vestibular Research Laboratory, School of Psychology, The University of Sydney, Darlington, NSW, Australia; ^2^MSA ENT Academy Center, Cassino, Italy

**Keywords:** vestibular, otolith, utricular, labyrinth, semicircular canal, VEMP = vestibular-evoked myogenic potential

## The Origin of VEMP Tests Using High Frequencies to Identify SCD

We fully agree with the statement by Noij et al. (1) that for the detection of semicircular canal dehiscence (SCD) “High frequency VEMP testing is superior to all other methods described to date. It is highly specific for the detection of SCD and may be used to guide decision-making regarding the need for subsequent CT imaging” (1). The ideal is a very fast, innocuous test rather than extended and uncomfortable tests such as determining the threshold for VEMPs. Patients with a dehiscence show larger VEMPs and lower VEMP thresholds to air conducted sound (ACS) and bone conducted vibration (BCV). Standard VEMP stimuli (e.g., 500 Hz short tone bursts) are not optimal for such testing as Noij and Rauch reported. However, we wish to make clear that Manzari et al. (2) were the first to show that for clinical diagnosis of SCD a stimulus of 4,000 Hz is such a very simple very fast test with excellent specificity. We reported a (very short) Brief Communication in *Otolaryngology and Head Neck Surgery* showing the ocular VEMP to high frequency tone burst stimuli (either ACS or BCV) (2) to 4,000 Hz stimuli constituted a fast, simple innocuous functional test with a 100% success in showing SCD in 22 patients with CT verified SCD and the absence of VEMPs in 22 healthy control subjects. The test consisted of 50 presentations of brief (7 ms) tone bursts of high frequency (4,000 Hz) stimuli at a rate of 4/s instead of the standard VEMP test frequency of 500 Hz. Thus, the 4,000 Hz test is very short—a total of only 50 stimulus presentations were given at 4/s so the whole test is complete in 13 s. The sensitivity and specificity of the test was 1.0 and thus, diagnostic accuracy of 100%. In other words, if a patient had an oVEMP response to 4,000 Hz then they had a CT verified SCD. In that group of 22 healthy subjects, none had an oVEMP to 4,000 Hz stimulation. Leonardo Manzari discovered this very simple test at his clinic in Cassino, Italy and validated it on his patients with CT verified SCD and healthy controls. Others have followed his example with minor changes.

Noij and Rauch state in relation to our 2013 paper: “The high frequency oVEMP study using healthy subjects as the control group described 22 patients with unilateral and 4 patients with bilateral SCD (30 ears in total), while only 22 ears were included in the analysis. It is unclear which ears were excluded and why (31).” p.6 and later “A serious limitation of both published high frequency oVEMP studies was that some ears were excluded from analysis.”

This is not a serious limitation of our study. There is a very simple explanation for the numbers in the Manzari et al. (2) study. The VEMP data graphed and included in the analysis were for the 22 patients with unilateral SCD. The data for 4 patients with bilateral SCD (8 ears) was not included for the very simple reason that for these 4 patients we could not be certain which ear was responsible for the VEMP- the patients had enhanced VEMPs beneath both eyes but there may have been a contribution of the ipsilateral ear to the ipsilateral oVEMP! All the other 22 patients were unilateral SCD so the oVEMP beneath the eye contralateral to the SCD ear uniquely identified it. Rather than include the results (8 ears) from these patients with bilateral SCD who constitute a different group, we chose the conservative approach of not including these data in the graphical and numerical analysis. Had the data from these patients been included then the number of SCD detections would have increased but the sensitivity and specificity cannot increase further because they cannot exceed 1.0!.

## The Explanation of VEMP Responses to High Frequencies After SCD

Noij and Rauch attribute the increased VEMP amplitude to the stimulus generating a stronger otolithic response after SCD. They state: “The 2 and 4 kHz sound stimuli are at the upper edge of the otolith organ tuning curve. Since the otolith organs are relatively insensitive to acoustic signals at these higher frequencies, vestibular activation produced by a high frequency sound stimulus is usually insufficient to provide consistent responses in normal healthy individuals. However, in the presence of a dehiscent superior semicircular canal, the otolith organ ‘sees' a much higher ‘dose' of stimulus energy due to the shunting effect of the third window, resulting in a highly reliable cVEMP (and oVEMP) response to high frequency stimuli in SCD patients.”

**Figure 1:**
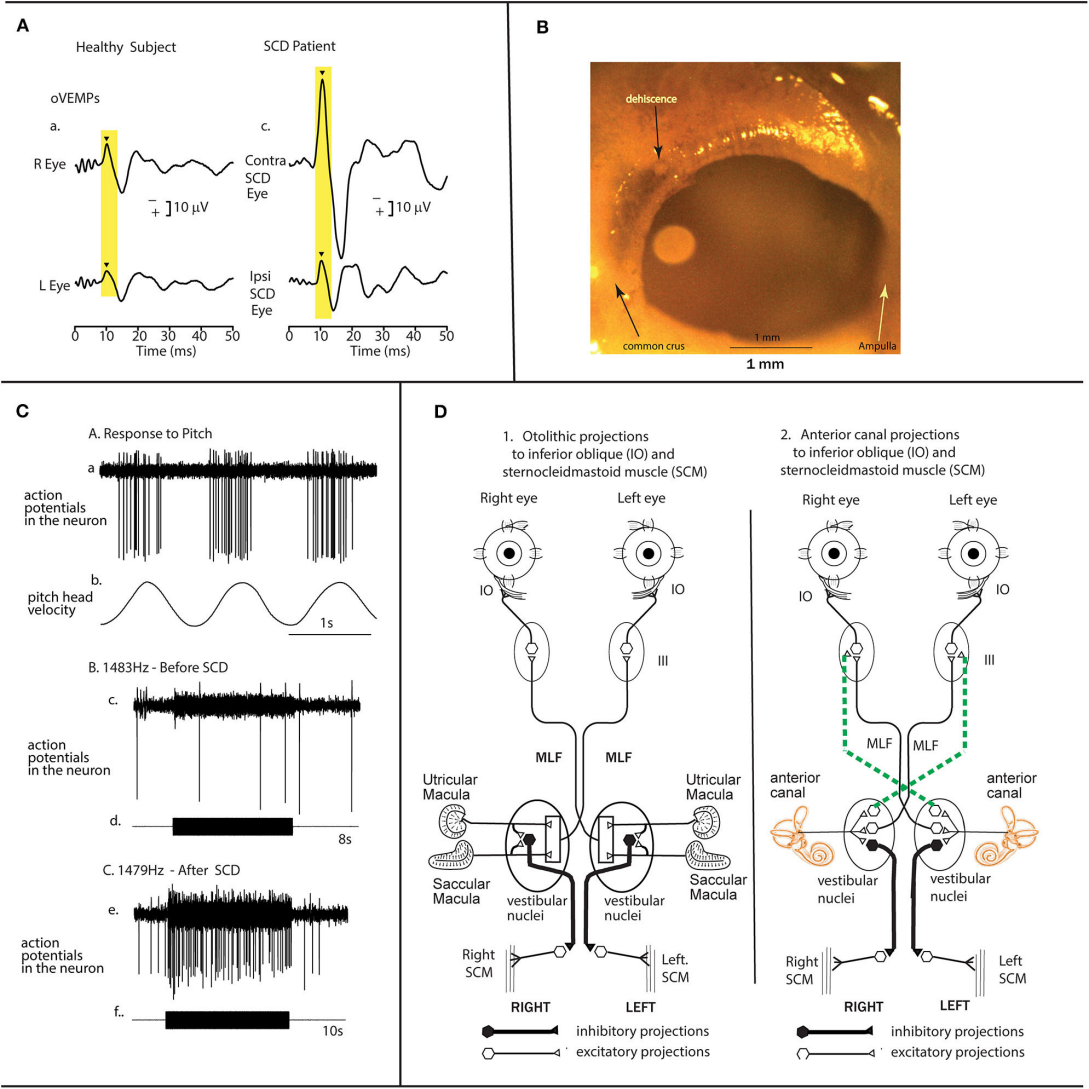
The anatomical and physiological basis for enhanced oVEMP responses after a semicircular canal dehiscence. **(A)** Time series records of the oVEMP response of a patient with an SCD showing a greatly enhanced oVEMP n10 component beneath the eye contralateral to the SCD in response to 500 Hz brief tone bursts. The typical oVEMP n10 response of a healthy subject to the same stimulus shows a much smaller n10 component [reproduced with permission from (4)]. **(B)** A view from a medial view point of the bony wall of the superior semicircular canal in a guinea pig showing the small (0.1 mm diameter) dehiscence of the bony wall of the canal made during the experiment by shaving away the thin bone using a fine scalpel blade, while continuing to record from the same single neuron. In guinea pigs the canal is clearly visible after removal of the overlying cerebellum [reproduced with permission from (5)]. **(C)** After such an SCD, anterior canal neurons with irregular resting activity are activated and phase-lock to ACS and BCV at stimulus levels used for human clinical testing, whereas they do not respond to the same stimuli before SCD (6). In these experiments the same neuron was tested before and after the SCD. The response of one superior canal neuron to high-frequency air-conducted sound, before and after a small dehiscence in the bony wall of the superior canal. (a) The response of the neuron to pitch angular acceleration in the plane of the superior canal identifies the neuron as being a superior canal afferent. (b) Before SCD an 8 s burst of 1,483 Hz ACS has no effect on the neural response—there are very few action potentials during the tone burst. (c) After the SCD a 10 s burst of an air-conducted sound of 1,479 Hz causes strong activation of this same neuron. Reproduced with the permission of John Wiley and Sons Inc., from (3). **(D)** The projections of otolithic (D1) and canal (D2) neurons to IO and SCM [redrawn from (7)]. These are schematic diagrams of a view of the brainstem to show the otolithic projections to IO and SCM on the left (1) and the superior canal projections to IO and SCM on the right (2). These projections were derived from experiments using electrical stimulation to identify the projections. Stimulation in animals with intact labyrinths causes the otolithic neural connections shown in 1 to be activated, so ACS and BCV generate the oVEMP and cVEMP responses without any input from semicircular canal neurons, since canal afferents are not activated by ACS and BCV at frequencies above 200 Hz (8). However, after an SCD, the otoliths are activated even more strongly but in addition superior semicircular canal neurons are also activated by ACS and BCV as well as the otolithic neurons. Some canal afferents can be activated by frequencies of 3,000 Hz and above (3) (9). The superior canal neurons project to III nucleus by the crossed ventral tegmental tract (dashed lines) and the MLF. This combination of otolithic and canal afferent activation will result in a larger oVEMP 10 (as shown in **A** above). Reproduced with the permission of John Wiley and Sons Inc., from (3).

This statement is correct: recording of single otolithic neurons before and after SCD shows that the SCD does cause enhanced otolithic neural response and that is true for both ACS and BCV stimuli (3) but it is only part of the reason for the enhanced response oVEMP response after SCD. Anatomy and physiology show there is another neural input contributing to the enhanced responses after SCD and clinicians should be aware of this. Superior canal afferent neurons project indirectly to both contralateral inferior oblique via the contralateral III nerve nucleus (the source of oVEMPs) and also to ipsilateral sternocleidomastoid muscle (the source of cVEMPs) (Figure 1D). High frequency ACS and BCV at clinically acceptable levels do not cause superior canal neurons to be activated in healthy animals if the labyrinth is encased in bone as it normally is (see Figures 1A–C). However, after an SCD these superior canal neurons are activated at low threshold by high frequency stimuli so high frequency stimuli used in clinical testing will cause a marked increase in neural firing of these superior canal neurons which will contribute to both oVEMP and cVEMPs and enhance both VEMPs [it must be noted that very recent evidence shows that in animals with normally encased labyrinths, superior canal afferent neurons can be activated by very low frequencies—less than about 200 Hz (8)].

The evidence for these statements comes from physiological studies. One previous paper had shown this response (10). So Curthoys undertook to confirm and extend the result: in mammals do identified semicircular canal neurons respond to such high frequencies after SCD? The simple answer is yes (5, 6, 9, 11–17). The approach in this work was to record the response of single primary vestibular neurons in guinea pigs to sound and vibration before, during, and after making a dehiscence in the superior semicircular canal (Figure 1B). The neurons were identified by their location in Scarpa's ganglion and by their response to angular accelerations in semicircular canal planes or to maintained tilts. These recordings show that superior semicircular canal neurons in healthy guinea pigs with the labyrinth encased in bone (as is normal) are not activated by high frequency ACS or BCV stimulation at levels used in clinical testing. However, after a dehiscence of the bony superior canal there is clear strong increase in neural firing to the same stimulus which was ineffectual before SCD (see Figure 1C). Recording from the same neuron before, during, and after shaving away the bone to make a small dehiscence (Figure 1B) is an extremely difficult procedure but it provides definitive evidence that superior canal neurons are activated by high frequency sound and vibration after an SCD, but not before. It was repeated in over 70 neurons with the same results. The neurons are activated and show phase locking to these high frequencies (5, 9). The physiological results show that when a dehiscence as small as 0.1 mm diameter is made in the bony wall of the superior canal is made (Figure 1A) it causes substantial response to sound and vibration. Recently others have corroborated these results in toadfish (18). These physiological results provide the neural basis for high frequency testing for identifying SCD.

We summarized it thus:

“After SCD the threshold for otolith neurons to ACS and BCV also drops. Compared to normal animals the same stimulus will recruit more otolithic afferent neurons, and superior canal neurons will now also be activated. The superior canal afferents project to contralateral inferior oblique and to ipsilateral SCM. So, after SCD the ACS or BCV stimulus will cause neural drive to these muscles from the superior canal in addition to the enhanced otolithic-IO response. That result explains many clinical phenomena—the enhanced VEMP responses to sound and vibration after an SCD in patients with an SCD, where stimuli with frequencies as high as 4,000 Hz cause oVEMPs and cVEMPs [(2, 3), p. 967].”

## Conclusion

In conclusion the very simple test that Leonardo Manzari discovered—adjusting the frequency of the audiometer delivering the VEMP stimulus to deliver 4,000 Hz instead of 500 Hz—is a very simple fast and innocuous way of identifying SCD and Manzari's primacy deserves due recognition. What appears to be a minor modification of the test stimulus is in fact an entirely new and very specific way of testing SCD and the results from anatomy and physiology show why.

## Author Contributions

IC wrote the paper and reviewed and approved the final version. LM reviewed and approved the final version. All authors contributed to the article and approved the submitted version.

## Conflict of Interest

The authors declare that the research was conducted in the absence of any commercial or financial relationships that could be construed as a potential conflict of interest.

## Abbreviations

ACS: air-conducted sound
BCV: bone-conducted vibration
SCD: semicircular canal dehiscence
VEMP: vestibular evoked myogenic potential
oVEMP: ocular vestibular evoked myogenic potential
cVEMP: cervical vestibular evoked myogenic potential.
